# Increased thrombin activatable fibrinolysis inhibitor activity is associated with hypofibrinolysis in dogs with sepsis

**DOI:** 10.3389/fvets.2023.1104602

**Published:** 2023-02-16

**Authors:** Katherine E. Sotos, Robert Goggs, Alyssa P. Stablein, Marjory B. Brooks

**Affiliations:** ^1^Department of Clinical Sciences, College of Veterinary Medicine, Cornell University, Ithaca, NY, United States; ^2^Comparative Coagulation Laboratory, Animal Health Diagnostic Center, Cornell University, Ithaca, NY, United States

**Keywords:** sepsis, antithrombin, antiplasmin, TAFI, fibrinogen, D-dimer, dogs, fibrinolysis

## Abstract

**Introduction:**

Disorders of coagulation are well-recognized in dogs with sepsis, but data regarding fibrinolysis disorders are limited. We aimed to characterize fibrinolysis in dogs with sepsis compared to healthy controls. We hypothesized that dogs with sepsis would be hypofibrinolytic, and that hypofibrinolysis would be associated with non-survival.

**Methods:**

This was a prospective observational cohort study. We enrolled 20 client-owned dogs with sepsis admitted to the Cornell University Hospital for Animals and 20 healthy pet dogs. Coagulation and fibrinolytic pathway proteins including antiplasmin activity (AP), antithrombin activity (AT), thrombin activatable fibrinolysis inhibitor activity (TAFI), D-dimer concentration, fibrinogen concentration, and plasminogen activity were measured and compared between groups. Overall coagulation potential, overall fibrinolysis potential, and overall hemostatic potential were calculated from the curve of fibrin clot formation and lysis over time.

**Results:**

Compared to healthy controls, dogs with sepsis had lower AT (*P* = 0.009), higher AP (*P* = 0.002), higher TAFI (*P* = 0.0385), and higher concentrations of fibrinogen (*P* < 0.0001) and D-dimer (*P* = 0.0001). Dogs with sepsis also had greater overall coagulation potential (*P* = 0.003), overall hemostatic potential (*P* = 0.0015), and lower overall fibrinolysis potential (*P* = 0.0004). The extent of fibrinolysis was significantly negatively correlated with TAFI. No significant differences were observed between survivors and non-survivors.

**Discussion:**

Dogs with sepsis were hypercoagulable and hypofibrinolytic compared to healthy dogs, suggesting potential utility of thromboprophylaxis in this patient population. The association between high TAFI and low overall fibrinolysis potential might provide a potential mechanism for this hypofibrinolysis.

## Introduction

Sepsis is a major cause of morbidity and mortality in dogs (1), and is defined as the dysregulated host response to infection that causes organ dysfunction (2), including disorders of the hemostatic system (3). Sepsis is associated with development of a procoagulant state (4, 5), that can manifest as disseminated intravascular coagulation (DIC) (6), perpetuating organ damage (7), and causing clinical thrombosis (8–11). This procoagulant state results from activity of proinflammatory cytokines (12–15), that induce de novo intravascular tissue factor expression (16, 17), and diminish concentrations of endogenous inhibitors and anticoagulants, including thrombin-activatable fibrinolysis inhibitor (TAFI), antithrombin (AT) and protein C (3, 5, 18).

Suppression of the fibrinolytic system is an important predictor of development of multiple organ dysfunction syndrome (MODS) and mortality from sepsis in humans (19–21). The mechanism of fibrinolytic dysregulation in sepsis is complex. Plasmin, the primary effector of fibrinolysis, is generated from cleavage of plasminogen by tissue plasminogen activators (tPA). Low plasminogen concentrations preceded development of thrombocytopenia in a case series of humans with sepsis (22), and have been described in dogs with DIC due to cancer, pancreatitis and sepsis (23). Increased tPA activity occurs during sepsis to promote fibrinolysis (21), however simultaneous upregulation of fibrinolysis inhibitors oppose this action (24). The primary inhibitor of tPA is plasminogen activator inhibitor-1 (PAI-1). PAI-1 is an acute phase reactant and increased PAI-1 activity has been documented in humans with sepsis (24, 25). These patients also have decreased antiplasmin (AP) activities (19), attributed to increased formation and clearance of plasmin-antiplasmin complexes (26–28). In humans with sepsis, alterations in fibrinolytic pathway proteins predict thrombocytopenia (22), and have prognostic value (29, 30). Fibrinolysis is also inhibited by the plasma carboxypeptidase, thrombin activatable fibrinolysis inhibitor (TAFI). Activated TAFI removes the carboxyl-terminal lysine residues from fibrin that promote plasminogen binding to fibrin and enhance plasmin-mediated fibrinolysis (21). In humans with sepsis, decreased TAFI activity is associated with organ dysfunction (31, 32), and consumption of TAFI is an independent predictor of mortality (29).

Fibrinolytic pathway proteins including AP, plasminogen and TAFI have been studied in dogs with babesiosis, cancer and endocrinopathies (33–35), but similar studies of fibrinolysis in dogs with sepsis have not been performed. Given the complexity of the coagulation system (36, 37), simultaneous analysis of multiple fibrinolysis proteins might aid assessment of the disturbances present in dogs with sepsis. The main objective of our study was to characterize fibrinolysis in dogs with sepsis through measurement of individual fibrinolytic pathway proteins, combined with assessment of a global test of fibrinolysis, referred to as overall hemostasis potential (OHP) (38, 39). We hypothesized that dogs with sepsis are hypofibrinolytic, have fibrinolysis profiles distinct from those of healthy dogs, and that hypofibrinolysis is associated with non-survival.

## Materials and methods

### Study design

This was a prospective observational cohort study of client-owned dogs with sepsis admitted to the Cornell University Hospital for Animals. Dogs were eligible for enrollment if they weighed >5 kg, had a documented clinical syndrome associated with systemic infection (such as pyometra, septic peritonitis or pneumonia) and satisfied ≥2 systemic inflammatory response syndrome (SIRS) criteria, specifically: hypo- or hyper-thermia, temperature <37.8 or >39.4°C (<100.0 or >102.9°F); tachycardia, heart rate >140 bpm; tachypnea, respiratory rate >20 bpm; leukopenia or leukocytosis, <6 × 10^3^/μL or >16 × 10^3^/μL or >3% band neutrophils (3, 40). When bacterial cultures were not performed, or were negative, sepsis was confirmed by alternative means e.g., confirmation of gastrointestinal content leakage, surgical lesion identification, or determination through radiographic criteria. Dogs were ineligible if they had sepsis due to viral disease e.g., parvovirus or fungal disease e.g., candidiasis. To protect enrolled animals from potential complications associated with venipuncture, dogs with severe anemia, coagulopathy, or thrombocytopenia (Hb <5 g/dL; PT or aPTT >150 % normal; platelets <30 × 10^3^/μL) were excluded. Dogs not expected to live more than 12 h were also ineligible. Dogs were enrolled with written informed client consent. The local Institutional Animal Care and Use Committee approved the study protocol (Cornell IACUC Protocol #2014-0053). Healthy dogs were recruited from staff-owned pets and were eligible for the study if they weighed >5 kg, were aged between 1 and 9 y, had no chronic or recent illness, and had received no medications other than preventative healthcare (e.g., parasiticides) in the preceding 3 months. Dogs were classified as healthy based on history, physical examination, and the results of complete blood count and serum biochemistry profile results.

### Case management and evaluation

Primary clinicians determined all aspects of case management. Signalment and physical examination findings at hospital admission were recorded. Blood gases, electrolytes and lactate concentrations were measured immediately after sample collection with a point-of-care device (RapidPoint 500, Siemens Healthcare, Malvern, PA). Complete blood counts (CBC) (ADVIA 2120, Siemens Healthcare) with clinical pathologist review and serum biochemistry profiles (Cobas C501, Roche Diagnostics, Indianapolis, IN) were analyzed immediately whenever possible, but always within 48 h of collection. Mentation score, blood glucose, albumin and lactate concentrations and platelet counts were used to calculate the acute patient physiologic and laboratory evaluation illness severity score (APPLE_fast_) (41, 42). Outcome status at discharge was recorded as survived, died, or euthanized. Blood samples were collected at study entry, and prior to administration of any antithrombotic medications, into evacuated tubes (Vacutainer, BD and Co, Franklin Lakes, NJ) containing no-additive (for serum biochemistry analyses), 3.2% sodium citrate (1:9 ratio) (for coagulation testing) and K_2_-EDTA (for complete blood counts). Citrate plasma was prepared from whole blood by centrifugation for 10 min at 1,370 g (Ultra-8V Centrifuge, LW Scientific, Lawrenceville, GA). Plasma was transferred into polypropylene freezer tubes (Polypropylene Screw-Cap Microcentrifuge Tubes, VWR, Radnor, PA) with some plasma deliberately left in each tube to minimize the risk of cell contamination and frozen at −80°C pending batch analysis.

### Coagulation and fibrinolysis testing

Determination of antiplasmin activity (AP), antithrombin activity (AT), D-dimer concentration, fibrinogen concentration (Clauss fibrinogen) and plasminogen activity was performed using an automated instrument with mechanical and spectrophotometric endpoint detection modes (STA Compact Max, Diagnostica Stago, Parsippany, NJ). Plasma AP and AT were measured with the manufacturer's synthetic chromogenic substrate kits (Stachrom Antiplasmin and Stachrom AT III, Diagnostica Stago). The AP assay is configured with a plasmin substrate and human plasmin reagent such that residual cleavage of the substrate is inversely proportional to AP in the test plasma. The AP standard curve was derived from a human plasma standard, with results reported as percentage of the human standard. The AT assay was modified using a pooled canine plasma standard (prepared at the Coagulation Laboratory from 20 healthy dogs). The pooled canine plasma had an assigned value of 100% AT and results were reported as percentage of the canine standard. Plasma D-dimer concentration was measured using a quantitative, turbidimetric immunoassay and the manufacturer's human D-dimer calibration standard (HemosIL D-dimer and Calibrator, Instrumentation Laboratory, Lexington, MA). Fibrinogen concentrations were measured *via* the Clauss method using a human thrombin reagent (STA Fibrinogen 100 U/mL, Diagnostica Stago) and the canine plasma standard. The fibrinogen content of the plasma standard was determined by gravimetric method (43). Concentrations of D-dimer and fibrinogen were reported as ng/mL and mg/dL, respectively. Plasminogen activity was measured based on cleavage of a chromogenic plasmin substrate (S-2251, Diapharma, West Chester, OH) following sample incubation with urokinase (Prospec, East Brunswick, NJ), as previously described (44). The assay was modified by an initial acidification/neutralization step and results were reported as the percentage plasminogen activity of a pooled canine plasma standard with an assigned value of 100%.

Quantitation of TAFI activity was performed as previously reported, with minor modifications (18). Briefly, canine TAFI activity was analyzed using a commercial kinetic chromogenic assay kit (Pefakit TAFI, Pentafarm, Basel, Switzerland). The assay uses a thrombin-thrombomodulin complex reagent to activate TAFI in the test plasma. Activated TAFI then acts on a synthetic chromogenic TAFI substrate. Plasma samples were diluted 1:2 in 0.9% sodium chloride prior to analysis. Diluted plasma samples were combined with the thrombin-thrombomodulin reagent in a 1:10 ratio in a 96-well microtiter plate and incubated for 3 min at 37°C before addition of the synthetic substrate. Upon addition of the substrate, the absorbance at 405 nm of each well was monitored every 10 s for 5 min in an automated plate reader (Cytation 1, BioTek Agilent, Santa Clara, CA). All measurements were run in duplicate. The activity of TAFI was expressed as percent activity of a pooled human plasma provided as a calibrator.

Fibrin clot formation and lysis over time in patient plasma was evaluated in the OHP assay (38, 39). The assay was configured with paired reaction mixtures containing test plasma and thrombin to generate a coagulation curve, and test plasma with thrombin and tPA to generate a fibrinolysis curve (39, 45). The assay was performed as previously described (45). In brief, coagulation and lysis reactions were performed in flat-bottom 96-well microtiter plates containing seventy-five microliters of plasma and a buffer containing bovine alpha-thrombin (final concentration 0.05 U/mL). The lysis reactions also contained human recombinant tPA (final concentration 350 ng/mL). After addition of the buffer containing coagulation and fibrinolysis activators to the test plasma, absorbance at 405 nm was measured every minute for 60 min and plotted over time to visualize changes in turbidity related to fibrin formation and degradation. The parameter overall coagulation potential (OCP) was defined as the area under the coagulation curve. This parameter is a measure of the rate and amount of fibrin formation. The OHP parameter was defined as the area under the curve in the lysis reaction containing thrombin and tPA, and thus depends on both fibrin formation and fibrinolysis. The overall fibrinolysis potential (OFP) parameter is derived from the OCP and OHP values. The OFP is calculated as the relative difference in area between the coagulation and lysis curves: OFP% = (OCP – OHP)/OCP × 100.

### Statistical methods

Continuous data (e.g., dog characteristics, physical examination findings and clinicopathologic values) were assessed for normality using the D'Agostino Pearson test and appropriate descriptive statistics calculated. Comparisons between groups were performed using unpaired *t*-tests with Welch's correction for normally distributed data or the Mann-Whitney U test when data were non-parametric. Correlations between coagulation parameters were evaluated using Spearman's correlation coefficients, associated *P*-values and scatterplots. Strength of correlation was assessed as follows: <0.5 weak, 0.5–0.6 mild, 0.6–0.7 moderate, 0.7–0.8 strong, 0.8–0.9 very strong, 0.9–1.0 excellent. The overall pattern of coagulation values in dogs with sepsis were compared with those in healthy dogs using radar plots (Excel for Mac, Microsoft, Redmond, WA). Coagulation variable data for each dog was ratioed against the midpoint of the corresponding reference interval. The within-group means of these fold values corresponding to the degree of divergence from the reference interval were then overlaid on a hexagonal reference chart to allow visual comparison of dogs with sepsis with healthy controls. Using comparisons against the relevant reference interval, the coagulation disturbances in each dog with sepsis was classified as hyperfibrinolytic, hypofibrinolytic, mixed disturbance, or no disorder. Dogs satisfying 3/5 of the following criteria: low TAFI, low fibrinogen, high D-dimer, low AP, and low plasminogen were classified as hyperfibrinolytic, most consistent with a bleeding risk. Dogs satisfying 3/5 of the following criteria: high TAFI, high fibrinogen, high AP, low AT, and high plasminogen were classified as hypofibrinolytic, most consistent with a thrombotic risk. Dogs with disturbances characteristic of more than one type of disturbance were categorized as having a mixed disorder. Dogs with 4/6 parameters within the reference interval were classified as no disorder. The association between coagulation status classification (hypofibrinolytic versus other) and outcome was assessed with Fisher's exact test. Statistical analyses were performed using commercial software (Prism 9 for macOS, GraphPad, La Jolla, CA) with alpha set at 0.05. No *post-hoc* corrections were made for multiple comparisons because all between group comparisons were based on *a priori* hypotheses.

## Results

### Animals

A total of 40 dogs were enrolled; 20 dogs with sepsis and 20 healthy controls. The 20 dogs with sepsis had a variety of different diseases, specifically four dogs had abscesses or cellulitis, three dogs had peritonitis, three dogs had pneumonia, three dogs had pyometra, and two dogs had mastitis. Other causes included anaplasmosis, gastroenteritis (with bacteremia), osteomyelitis, pyothorax and urosepsis (all *n* = 1). Of the 20 dogs, three were euthanized for disease severity prior to discharge, the remainder survived to discharge, equivalent to a 15% case fatality rate. Of the 17 dogs that survived to hospital discharge, 16 dogs were alive at day 28, with 1 dog lost to follow up, equivalent to a 16% 28-day case fatality rate. Demographic characteristics, initial assessments and clinicopathologic variables are summarized in Table 1. Dogs had been treated with a variety of medications prior to study enrolment, summarized in Table 2. Positive cultures were obtained in 64% (9/14) dogs for which culture samples were submitted. Bacterial organisms cultured from the dogs included *Escherichia coli* (*n* = 4), *Staphylococcus pseudintermedius* (*n* = 2), *Actinomyces canis, Bacteroides* spp*., Clostridium perfringens, Enterococcus faecium, Fusobacterium* sp., *Microbacterium phyllosphaerae, Mycoplasma* sp., *Peptostreptococcus* sp., and *Pseudarthrobacter* sp. (all *n* = 1).

**Table 1:** Summary of population characteristics including complete blood count and serum biochemistry data from study entry for dogs with sepsis in both SI and US units.

**Variable (SI units)**	**Dogs with sepsis (*n* = 20)**	**Healthy controls (*n* = 20)**	**Variable (US units)**	**Dogs with sepsis (*n* = 20)**
Age (y)	4.5 ± 3.5	4.6 ± 2.6	Age (y)	4.5 ± 3.5
Bodyweight (kg)	27.2 ± 15.1	33.9 ± 12.3	Bodyweight (kg)	27.2 ± 15.1
Sex (F/FS/M/MC)	6 5/4/5	0/13/1/6	Sex (F/FS/M/MC)	6/6/4/5
T (°C)	39.4 (38.3–40.0)		T (°F)	103 (101–104)
HR (bpm)	143 ± 21		HR (bpm)	143 ± 21
RR (bpm)	31 (26–37)		RR (bpm)	31 (26–47)
SAP (mmHg)	139 ± 33		SAP (mmHg)	139 ± 33
MAP (mmHg)	108 ± 27		MAP (mmHg)	108 ± 27
DAP (mmHg)	93 ± 28		DAP (mmHg)	93 ± 28
SpO_2_ (%)	95 ± 3		SpO_2_ (%)	95 ± 3
SIRS criteria (n)	3 (2–3) [Max 4]		SIRS criteria (n)	3 (2–3) [Max 4]
APPLE_fast_ score	21 (17–26) [Max 50]		APPLE_fast_ score	21 (17–26) [Max 50]
LoH (d)	3.5 (2–5)		LoH (d)	3.5 (2–5)
Lactate (mmol/L)	2.0 (1.3–3.5)		Lactate (mmol/L)	2.0 (1.3–3.5)
BG (mmol/L)	5.3 (4.4–6.3)	5.4 (5.0-5.7) [3.8–5.8]	BG (mg/dL)	97 (82–113) [68–104]
HCT (%)	45 ± 8.2 [41–58]	51 ± 6.3 [41–58]	HCT (%)	45 ± 8.2 [41–58]
Leukocytes (×10^9^/L)	16.9 ± 8.1 [5.7–14.2]	8.4 ± 4.0 [5.7–14.2]	Leukocytes (×10^3^/μL)	16.9 ± 8.1 [5.7–14.2]
Neutrophils (×10^9^/L)	11.7 ± 8.0 [2.7–9.4]	5.2 ± 3.2 [2.7–9.4]	Neutrophils ( × 10^3^/μL)	11.7 ± 8.0 [2.7–9.4]
Bands (×10^9^/L)	1.5 (0.3–3.7) [0.0–0.1]	0.0 (0.0–0.0) [0.0–0.1]	Bands ( × 10^3^/μL)	1.5 (0.3–3.7) [0.0–0.1]
Lymphocytes (×10^9^/L)	1.1 (0.5–3.0) [0.9–4.7]	2.0 (1.3–2.3) [0.9–4.7]	Lymphocytes ( × 10^3^/μL)	1.1 (0.5–3.0) [0.9–4.7]
Monocytes (×10^9^/L)	1.0 (0.5–2.0) [0.1–1.3]	0.3 (0.3-0.5) [0.1-1.3]	Monocytes ( × 10^3^/μL)	1.0 (0.5–2.0) [0.1–1.3]
Eosinophils (×10^9^/L)	0.0 (0.0–0.1) [0.1–2.1]	0.5 (0.3–0.7) [0.1–2.1]	Eosinophils (×10^3^/μL)	0.0 (0.0–0.1) [0.1–2.1]
Platelets (×10^9^/L)	219 (111–273) [186–545]	234 (191–274) [186–545]	Platelets (×10^3^/μL)	219 (111–273) [186–545]
Albumin (g/L)	25 (22–31) [32–41]	39 (37–40) [32–41]	Albumin (g/dL)	2.5 (2.2–3.1) [3.2–4.1]
ALT (U/L)	50 (26–110) [17–95]	45 (36–59) [17–95]	ALT (U/L)	50 (26–110) [17–95]
Total bilirubin (μmol/L)	1.7 (1.7–6.8) [0.0–3.4]	0.0 (0.0–0.0) [0.0–0.2]	Total bilirubin (mg/dL)	0.1 (0.1–0.4) [0.0–0.2]
BUN (mmol/L)	4.6 (3.2–7.9) [3.2–9.3]	6.1 (5.4–7.1) [3.2–9.3]	BUN (mg/dL)	13 (9–22) [9–26]
Creatinine (μmol/L)	106 ± 111 [53–124]	97 (80–97) [53–124]	Creatinine (mg/dL)	1.2 ± 1.3 [0.6–1.4]

Data are presented as mean ± standard deviation for normally distributed data and median (interquartile range) for non-normally distributed data. Values in square parentheses are the local laboratory reference intervals. Values for healthy control dogs are displayed for comparison.

**Table 2:** Medications prescribed to dogs with sepsis prior to study enrolment.

**Medication class**	***n***
Antimicrobial drugs (AMD) - Beta-lactams (*n* = 10) - Fluoroquinolones (*n* = 6) - Nitroimidazoles (*n* = 5) - Tetracyclines (*n* = 1) - Unknown AMD (*n* = 1)	23
Antiemetics/Gastroprotectants	7
Non-steroidal anti-inflammatory drugs	6
Other analgesics	2
Glucocorticoids	1
Anxiolytics	1

### Coagulation and fibrinolysis assays

**Figure 1:**
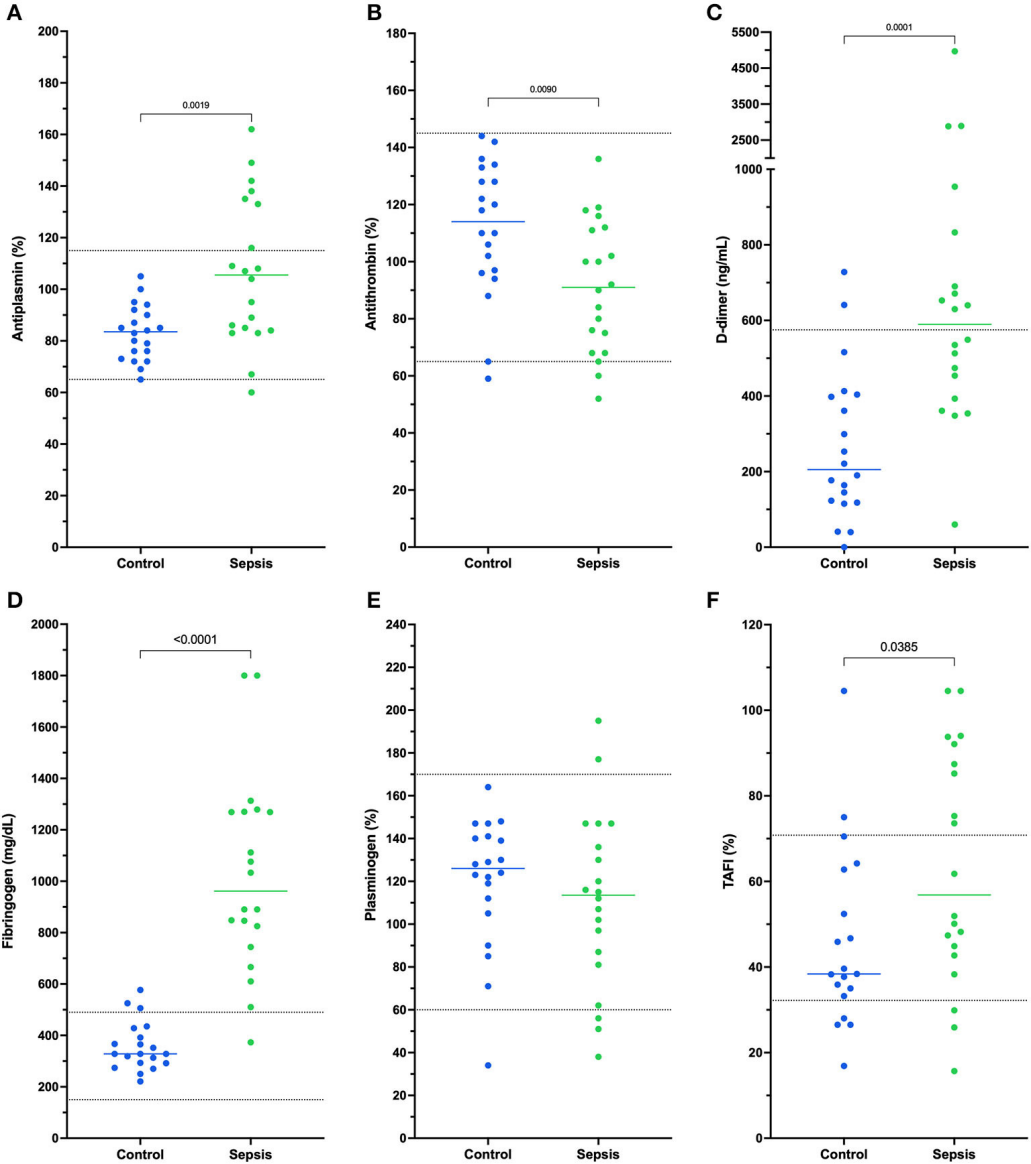
Dotplots of the activities of **(A)** antiplasmin (%), **(B)** antithrombin (%), concentrations of **(C)** D-dimer (ng/mL) and **(D)** fibrinogen (mg/dL) and activities of **(E)** plasminogen (%), and **(F)** thrombin-activatable fibrinolysis inhibitor (TAFI, %) in dogs with sepsis compared to healthy controls. Comparisons were performed using unpaired *t*-tests with Welch's correction or the Mann-Whitney U-test as appropriate based on data distribution. Compared to healthy controls, dogs with sepsis have significantly increased activities of antiplasmin and TAFI, significantly decreased antithrombin activity, and significantly increased concentrations of fibrinogen and D-dimer. Horizontal dotted lines represent laboratory reference interval bounds.

**Figure 2:**
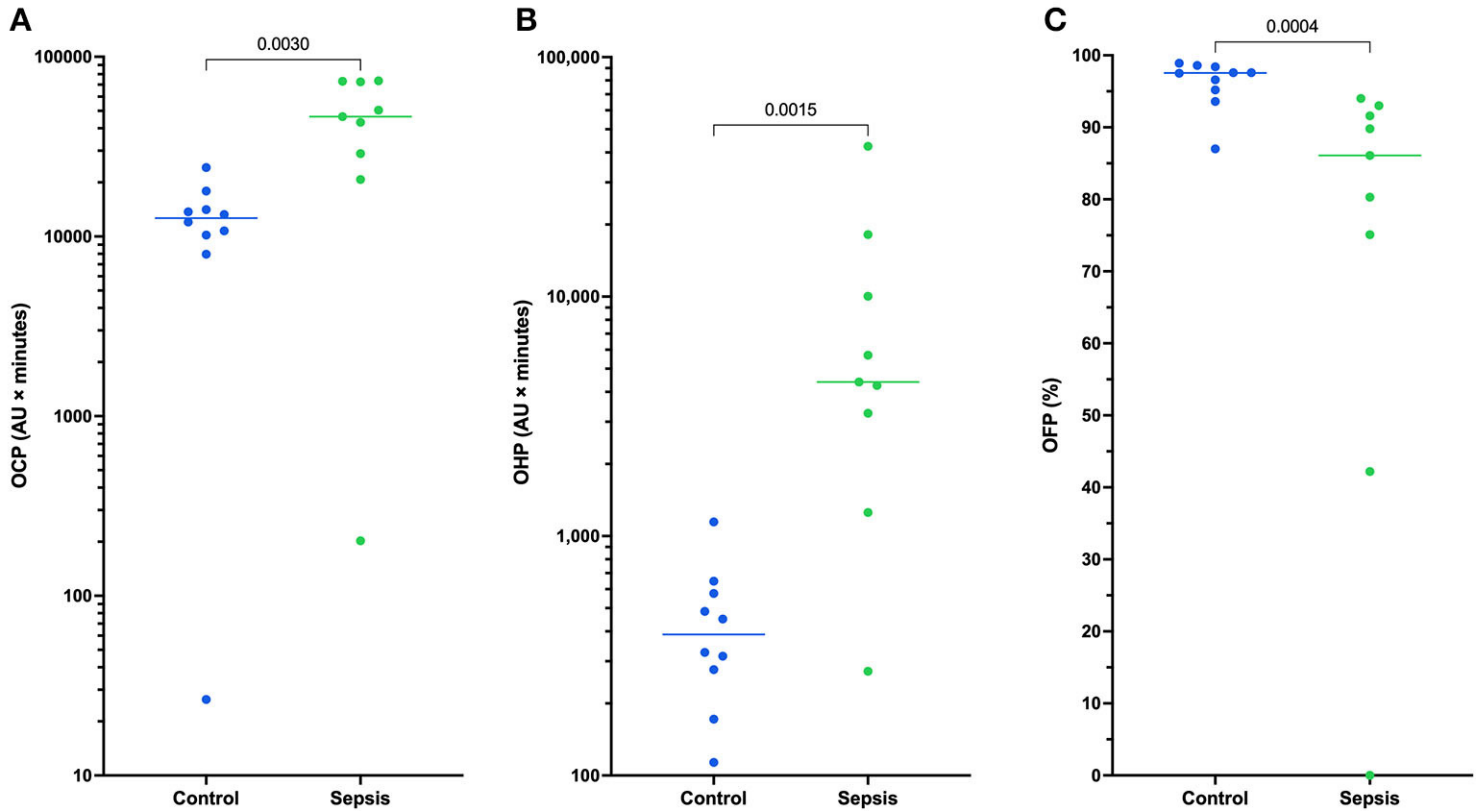
Dotplots of **(A)** the overall coagulation potential (OCP), **(B)** overall hemostatic potential (OHP), and **(C)** overall fibrinolytic potential (OFP) for 9 dogs with sepsis compared to controls (*n* = 10). Comparisons were performed using unpaired *t*-tests with Welch's correction or the Mann-Whitney U-test as appropriate based on data distribution. Dogs with sepsis have significantly greater OCP and OHP compared to healthy control dogs, and significantly lower OFP. This suggests dogs with sepsis have a greater propensity for clot formation and are hypofibrinolytic compared to control dogs. For 11 dogs with sepsis there was insufficient plasma sample volume available to perform the OHP/OFP assay.

**Figure 3:**
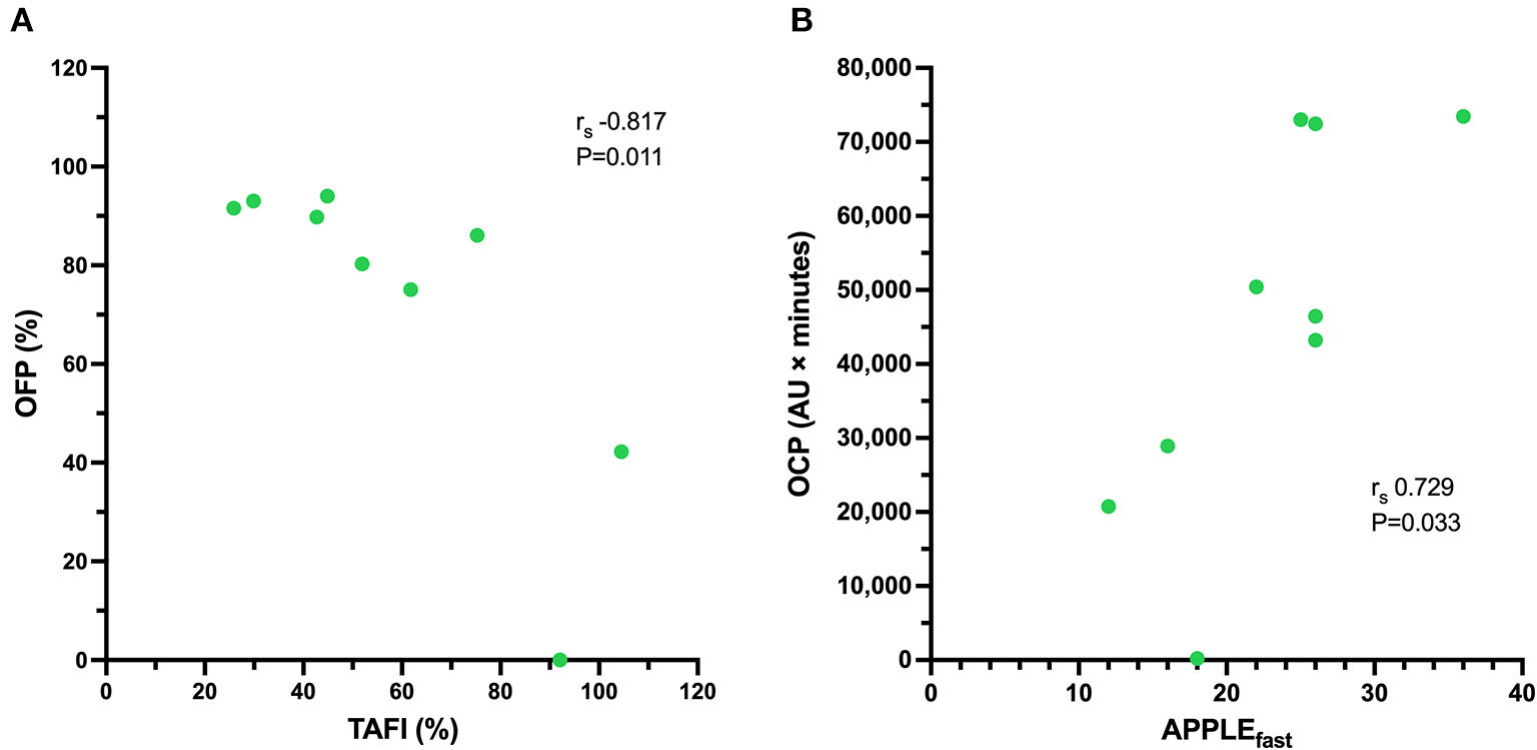
Scatterplot of **(A)** the activity of thrombin-activatable fibrinolysis inhibitor (TAFI, %, abscissa) against the overall fibrinolysis potential (OFP, %, ordinate) for nine dogs with sepsis. There is a significant and very strong negative correlation between the activity of TAFI and the OFP. Scatterplot of **(B)** the illness severity score (APPLE_fast_, abscissa) against the overall coagulation potential (OCP, AU × minutes, ordinate) for nine dogs with sepsis. There is a significant, strong positive correlation between illness severity and the OCP. For the remaining 11 dogs with sepsis there was insufficient plasma sample volume available to perform the OHP/OFP assay.

**Figure 4:**
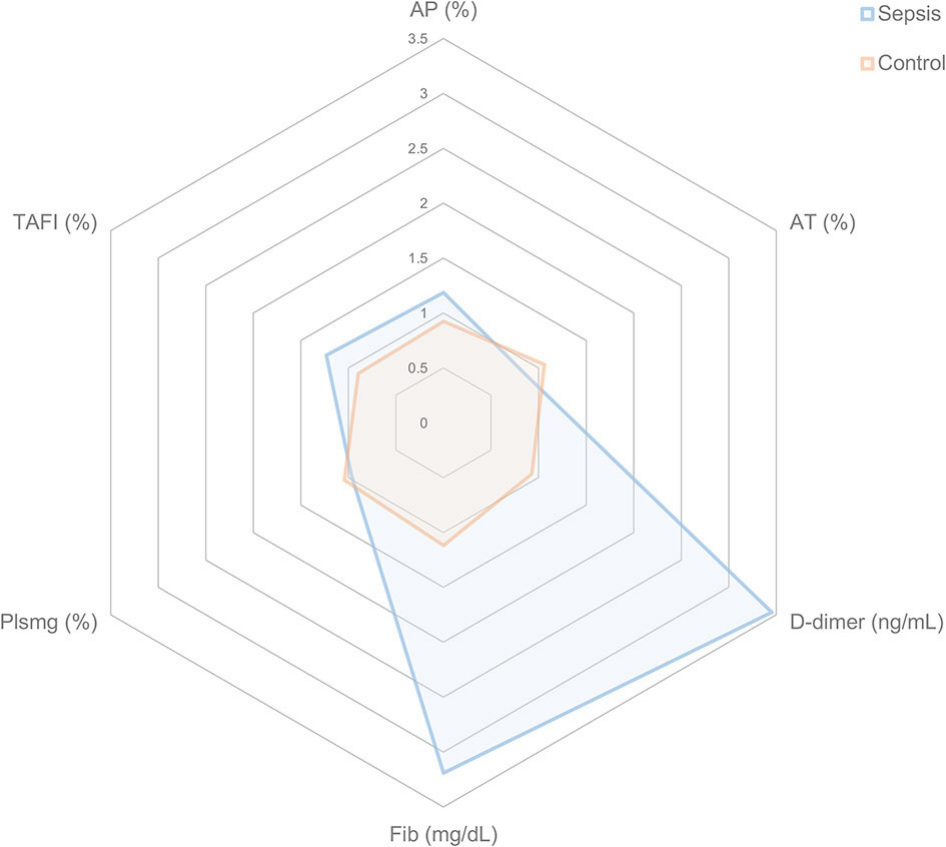
Radar plots representing the coagulation disturbances in dogs with sepsis compared to healthy control dogs. For each coagulation variable and for each individual dog, the fold change from the midpoint of the reference interval was calculated for dogs with sepsis and for healthy controls. The mean fold change for each coagulation variable was then plotted. A value of 1.0 indicates no mean deviation from the midpoint of the reference interval, while a value of 3.0 indicates there was a mean 3-fold increase above the midpoint of the reference interval for that variable. Consistent with their healthy, normal status, the mean values for control dogs are all close to 1.0. By contrast, dogs with sepsis have large mean deviations for D-dimer and Fib in particular. AP, antiplasmin; AT, antithrombin; Fib, fibrinogen; Plsmg, plasminogen; TAFI, thrombin-activatable fibrinolysis inhibitor.

All coagulation and fibrinolysis test results are summarized in Table 3. Compared to healthy controls, dogs with sepsis had significantly lower AT, higher AP and TAFI, and higher concentrations of clottable fibrinogen and D-dimer. No difference in plasminogen activity was observed between the two groups (Figure 1). Control samples on two OHP assay plates generated inappropriately low OHP values, indicating an assay error. Study samples quantitated on these plates were excluded from subsequent analyses and lack of sample volume precluded repeating these tests. Comparison of OHP assay results for the remaining septic dogs (*n* = 9) and controls (*n* = 10), revealed that septic dogs had significantly greater OHP, OCP and smaller OFP compared to healthy controls (Figure 2). Only two pairs of variables were associated with a Spearman correlation coefficient >0.7 (strong or better). The activity of the fibrinolysis inhibitor, TAFI, was negatively correlated with the extent of fibrinolysis, measured as % OFP (*r*_s_ −0.817, *P* = 0.011, Figure 3A), and the OCP was positively correlated with illness severity as assessed by APPLE_fast_ score (*r*_s_ 0.729, *P* = 0.033, Figure 3B). A single graphical display of the 6 individual tests revealed that fibrinogen and D-dimer concentrations in the septic dog group demonstrated the most profound deviations from reference intervals (Figure 4). No significant differences between the test values of septic dog survivors and non-survivors were observed (Supplementary material S1). Similarly, none of the coagulation variables were significantly associated with duration of hospitalization. Classification of dogs with sepsis based on the number of test parameters outside of their corresponding reference intervals suggested that seven dogs had a mixed disorder, seven dogs had no disorder, five dogs appeared hypofibrinolytic, and one dog appeared hyperfibrinolytic. There was no association between hypofibrinolytic status and non-survival to hospital discharge, relative risk 1.56 (0.97–4.07), *P* = 0.140.

**Table 3:** Descriptive statistics summarizing the coagulation and fibrinolysis test results from dogs with sepsis compared to healthy controls.

**Variable**	**Sepsis (*n* = 20)**	**Controls (*n* = 20)**	***P*-value**
Antiplasmin activity (%)	107 ± 29	83 ± 11	0.0019
Antithrombin activity (%)	91 ± 23	112 ± 24	0.0090
Fibrinogen (Clauss) (mg/dL)	1,021 ± 380	358 ± 95	<0.0001
D-dimer (ng/mL)	590 (408–797)	206 (119–403)	0.0001
Overall coagulation potential OCP (OD × min)	46,446 (24,834–72,740) ^(n = 9)^	12,642 (9,634–15,066) ^(n = 10)^	0.0030
^1^Overall fibrinolysis potential OFP (%)	86 (59–92) ^(n = 9)^	98 (95–98) ^(n = 10)^	0.0004
Overall hemostatic potential OHP (OD × min)	4,399 (2,255–14,105) ^(n = 9)^	388 (251–594) ^(n = 10)^	0.0015
Plasminogen (%)	114 (83–144)	126 (107–141)	0.3234
Thrombin-activatable fibrinolysis inhibitor (%)	57 (43–91)	38 (33–63)	0.0385

Data are presented as mean ± standard deviation for normally distributed data and median (interquartile range) for non-normally distributed data. *P*-values represent the results of between group comparisons conducted using unpaired *t*-tests with Welch's correction for parametric data and Mann-Whitney U tests for nonparametric data. Note that the numbers of dogs for which overall hemostatic potential assay data were available is lower than for other variables.

^1^Overall fibrinolysis potential OFP % = (OCP-OHP/OCP) ^*^ 100.

## Discussion

We aimed to characterize the fibrinolytic system in dogs with sepsis. Although there was some heterogeneity in the study population, dogs with sepsis were hypercoagulable and hypofibrinolytic relative to normal dogs. Depressed fibrinolysis combined with increased fibrinogen thus contributes to a prothrombotic risk in dogs with sepsis, as it does in humans (46, 47). Our results support recommendations that sepsis represents a risk factor for thrombosis in dogs that can warrant thromboprophylaxis (48, 49).

We observed numerous significant differences in the activities and concentrations of pro- and anti-fibrinolytic proteins in dogs with sepsis compared to healthy controls, most consistently increased fibrinogen and D-dimer concentrations. Concentrations of the fibrinolysis inhibitor TAFI were also significantly increased in dogs with sepsis and were very strongly correlated with the OFP. This association provides potential mechanistic insight, because high TAFI concentrations were correlated with low OFP values. High TAFI activity prevents tPA from colocalizing with plasminogen on fibrin thereby limiting plasmin generation and suppressing fibrinolysis. Increased TAFI concentrations are associated with increased risk of venous thrombosis and stroke in humans (50–52), although typically TAFI concentrations are either unchanged (53, 54), or decreased in humans with sepsis (55, 56). High TAFI concentrations were reported in dogs with babesiosis (33), and in dogs with sepsis (18), with concentrations comparable with those we observed. The cause of high TAFI concentrations in dogs with sepsis is uncertain. Increased hepatic synthesis of TAFI combined with decreased endothelial expression of thrombomodulin might result in a relative imbalance between production and consumption, thereby favoring higher plasma TAFI concentration (5, 57).

The increased AP activities observed in dogs with sepsis likely resulted from an acute phase response and further contributed to the hypofibrinolytic phenotype. In dogs, AP activity is also increased following minor and major surgery (58, 59), postoperative hemorrhage (60), and with protein-losing disease (61). AP is a serine-protease inhibitor (serpin) produced by the liver that binds plasmin, leading to its own cleavage and the subsequent formation of inactive antiplasmin-plasmin complexes, thereby limiting fibrinolysis (62). Additionally FXIIIa cross-links AP into growing thrombi (63), slowing plasmin-mediated clot lysis (64). AP may help prevent hyperfibrinolysis, and studies in various human diseases suggests that increased AP concentrations potentiate and perpetuate pathologic thrombi causing stroke, deep vein thrombosis and pulmonary embolism (65).

Prior studies have demonstrated that dogs with sepsis have increased fibrinogen concentrations consistent with the acute phase, evidence of consumption of the endogenous anticoagulants AT and protein C, and increased concentrations of D-dimers indicating fibrin degradation (3, 66, 67). Consistent with these reports, we identified significantly decreased AT and significantly increased fibrinogen and D-dimer concentrations. Dogs in our study had evidence of thrombin generation (decreased AT) and activation of the fibrinolytic system (increased D-dimer), but the increased fibrinogen concentrations do not suggest that a consumptive coagulopathy or sufficient criteria for diagnosis of overt DIC occurred in any dog (6). Rather, the typical phenotype of dogs in our study was consistent with inflammation, hypercoagulability and hypofibrinolysis. The OHP assay parameters OHP and OCP were increased, consistent with the increased fibrinogen concentration. Notably, the balance of fibrin formation and lysis described by the OFP parameter was low in septic dogs, confirming hypofibrinolysis. Assays of the individual components of the pathway suggest reduced plasmin generation and there may also be a structural explanation for the observed hypofibrinolysis. Thrombi formed when the initial rate of thrombin generation is high are dense, formed of tightly packed thin fibrin fibers that resist fibrinolysis, while those formed by lower rates of thrombin generation are looser with fibers that are coarse and more readily degraded by plasmin (68). This ultrastructural phenomenon may have *in vivo* consequences (69), and suggests that in the future, concurrent measurements of thrombin generation and plasmin generation potential could offer further insights into the pro-vs. anti-thrombotic balance present in patients *in vivo* (70).

There are limitations to the present study. The sample size in our study was small, restricted by costs and sample volumes required to perform the panel of coagulation and fibrinolysis assays. Low sample size may have increased the rates of type I and type II errors (e.g., for survival analyses) and precluded identification of discrete sub-populations. In addition, we did not evaluate every protein involved in the fibrinolytic system (47), in part due to lack of available or applicable assays. For instance, we measured plasminogen and AP, but did not determine the concentration of plasmin-antiplasmin (PAP) complexes or plasmin generation itself. Plasmin generation assays (70, 71) are in development but are presently not readily available for clinical studies in dogs (72). Similarly, PAP complexes have been assessed in humans with sepsis (26, 73), but a canine PAP assay is not presently available. Finally, assays to measure tPA and PAI-1 were unavailable (35), and might have provided valuable insights into the regulation of fibrinolysis in dogs with sepsis.

Most dogs in the study survived, likely due to low overall illness severity, moreover all three deaths were due to euthanasia, which could have biased survival analyses. As with all studies measuring blood biomarker concentrations, plasma levels of precursor coagulation proteins, their activated forms and their inhibitors are dynamic over time and are affected by fluid administration, blood product transfusion, disease-specific treatment, and administration of drugs such as aminocaproic acid and tranexamic acid that may directly influence the fibrinolytic system. Concurrent conditions including neoplasia, trauma, and liver disease and the effects of surgical interventions will inevitably affect the measured concentrations of pro- and antifibrinolytic proteins and *in vivo* hemostatic balance. Our study provides only a single time point observation precluding assessment of temporal changes or determination if the abnormalities identified represented the maximal disturbances associated with the disease. Experimental sepsis models enable sequential observations at defined times within the course of the syndrome, but do not replicate all features of naturally occurring disease.

In summary dogs with sepsis were hypercoagulable and hypofibrinolytic, characterized by increased plasma AP, D-dimer, fibrinogen and TAFI, and relative suppression of clot lysis in a global hemostasis assay, the OHP. These results support an increased potential for thrombotic complications in this patient population. Monitoring changes over time in the most readily available of these assays, such as fibrinogen and D-dimer, might provide guidance on individual patient risk and enable clinicians to individualize therapy with antithrombotic drugs. Future studies might include analyses of individual fibrinolytic pathway proteins, particularly tPA and PAI-1 and activated TAFI (53), and global assays, such as thrombin and plasmin generation, and viscoelastic measures of fibrinolysis to assess the overall balance of the fibrinolytic system (74, 75). Comparisons of the coagulation disturbances between underlying causes of sepsis might determine if all dogs with sepsis are comparably affected thereby improving our understanding of the causes of the coagulation dysfunction in dogs with sepsis. Identifying the underlying causes of hypofibrinolysis in dogs with sepsis through mechanistic studies (76, 77), might enable alternative therapeutic strategies to be employed in the future (78, 79).

## Data availability statement

The original contributions presented in the study are included in the article/Supplementary material, further inquiries can be directed to the corresponding author.

## Ethics statement

The animal study was reviewed and approved by the local Institution Animal Care and Use Committee (Protocol #2014-0053). Written informed consent was obtained from the owners for the participation of their animals in this study.

## Author contributions

KS recruited and enrolled patients, collected and analyzed data, and co-wrote the manuscript. RG conceived the study, analyzed data, and co-wrote the manuscript. AS collected and analyzed data and edited the manuscript. MB collected and analyzed data and edited the manuscript. All authors contributed to the article and approved the submitted version.
