# Supplementary figures and images for: Increased thrombin activatable fibrinolysis inhibitor activity is associated with hypofibrinolysis in dogs with sepsis
Source: Front Vet Sci. 2023 Feb 16;10:1104602. doi: 10.3389/fvets.2023.1104602 (PMC9978197; doi:10.3389/fvets.2023.1104602)

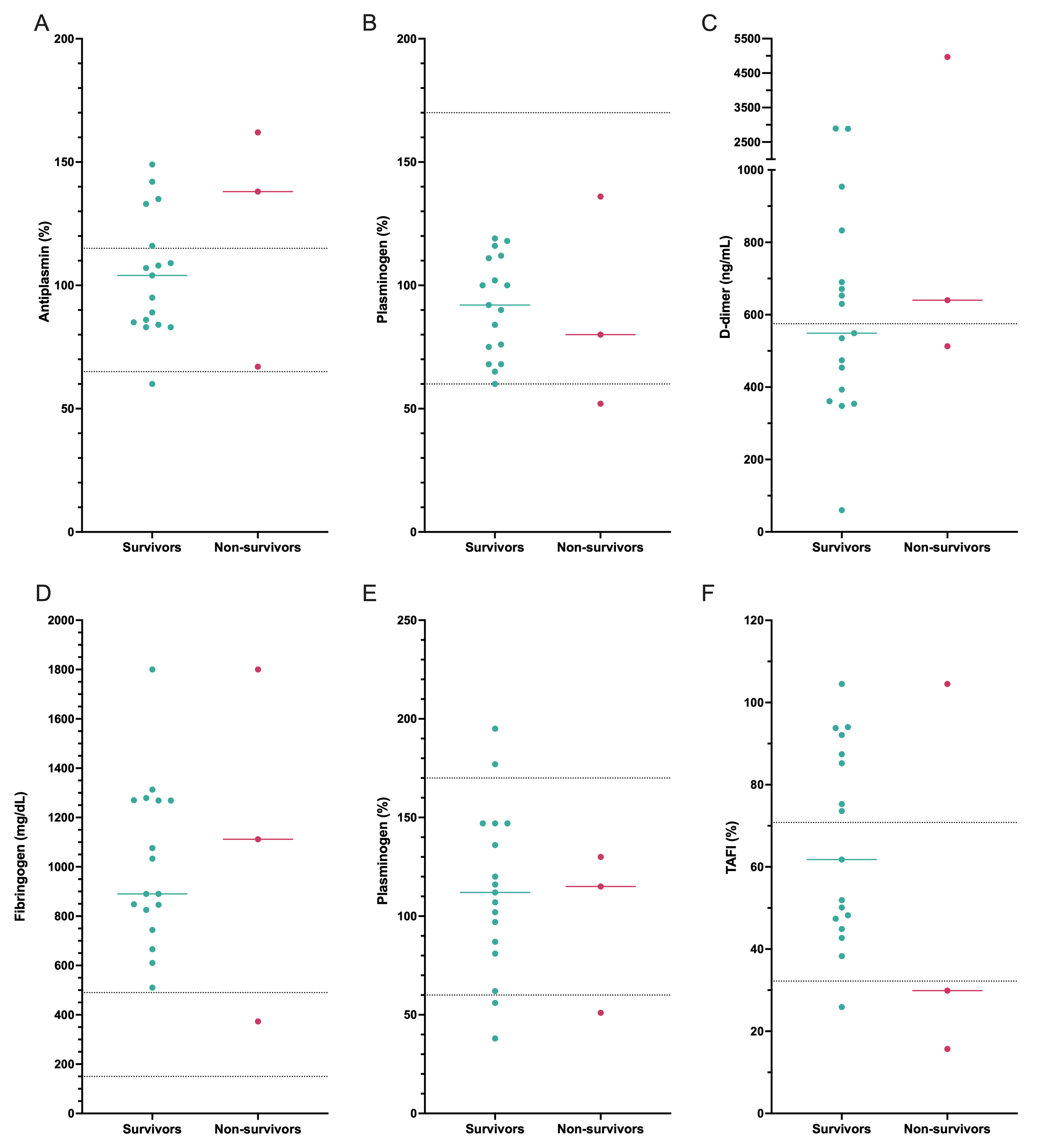

Supplement: Supplementary file 1 [file Image_1.tiff]
